# EEG Responses to Auditory Stimuli for Automatic Affect Recognition

**DOI:** 10.3389/fnins.2016.00244

**Published:** 2016-06-10

**Authors:** Dirk T. Hettich, Elaina Bolinger, Tamara Matuz, Niels Birbaumer, Wolfgang Rosenstiel, Martin Spüler

**Affiliations:** ^1^Developmental Aspects of Sleep, Memory, and Emotion, Institute of Medical Psychology and Behavioural Neurobiology, University of TübingenTübingen, Germany; ^2^Neural Interfaces and Brain Signal Decoding, Wilhelm-Schickard-Institute for Computer Science, University of TübingenTübingen, Germany; ^3^Ospedale San Camillo, IRCCSVenezia, Italy

**Keywords:** affective computing, brain-computer interface, event-related potential, late positive potential, machine learning, classification, support vector machine

## Abstract

Brain state classification for communication and control has been well established in the area of brain-computer interfaces over the last decades. Recently, the passive and automatic extraction of additional information regarding the psychological state of users from neurophysiological signals has gained increased attention in the interdisciplinary field of affective computing. We investigated how well specific emotional reactions, induced by auditory stimuli, can be detected in EEG recordings. We introduce an auditory emotion induction paradigm based on the International Affective Digitized Sounds 2nd Edition (IADS-2) database also suitable for disabled individuals. Stimuli are grouped in three valence categories: unpleasant, neutral, and pleasant. Significant differences in time domain domain event-related potentials are found in the electroencephalogram (EEG) between unpleasant and neutral, as well as pleasant and neutral conditions over midline electrodes. Time domain data were classified in three binary classification problems using a linear support vector machine (SVM) classifier. We discuss three classification performance measures in the context of affective computing and outline some strategies for conducting and reporting affect classification studies.

## 1. Introduction

Affective states consisting of emotions, feelings, and moods, are key in personal and interpersonal everyday life. Expressing and understanding emotions not only influences cognitive processes and therefore behavior, but also secures and maintains individual well-being (Damasio, 2004), particularly by enhancing the quality of communication. Classic human-computer interaction (HCI) lacks affect as a communication channel. Affective computing, i.e., computing that relates to, arises from, or influences emotions (Picard, 1995), seeks to improve HCI by including psychophysiological information (e.g., from brain signals, heart rate, or skin conductance), into HCI signal processing and evaluation (Fairclough, 2009). Assumptions about the relationship between elements of the physiological and psychological domains (Cacioppo et al., 2000) as well as valid machine learning approaches are crucial pitfalls in affective computing and its experimental design. Clinically, disabled or paralyzed individuals lacking oral communication could benefit the most from extending brain-computer interfaces (BCIs) for communication and control (Birbaumer et al., 1999; Wolpaw et al., 2002) by automatic affect recognition (Nijboer et al., 2009; Zander and Kothe, 2011; Mohamad et al., 2014).

Two main theories of emotion useful for BCI applications have been developed over the years: discrete emotion theory and dimensional emotion theory (Russell, 2009; Hamann, 2012). Discrete emotion theory suggests that humans express emotions based on on the combination of six basic emotions (happiness, sadness, anger, fear, disgust, and surprise) and that these emotions are universal, inherited and physiologically distinguishable from one another (Darwin, 1872; Ekman et al., 1983). Dimensional emotion theory, originating from the model by Wundt (1894), on the other hand, proposes that emotions are largely explained by the dimensions valence and arousal (Russel, 1980). Valence is whether the emotion is subjectively felt as positive or negative, and arousal is the energetic activation associated with the emotion. Linearly scaled valence and arousal (sometimes the dimension “dominance” is included) span a two-dimensional space covering a wide range of discrete emotions. Consequently, discrete emotional responses can coherently be grouped into categories, e.g., an unpleasant, a neutral, or a pleasant category regarding valence. In the context of brain signal-based affect recognition, the dimensional emotion theory has some benefits: the number of possible emotional labels can be held small.

The past decades have produced many findings about the electrophysiology of valence and arousal in emotional responses, leading to a number of EEG features that could possibly be utilized by affective HCI. In the time domain, affective stimuli were found to mainly influence component amplitudes of event-related potentials (ERPs). Researchers have reported conflicting evidence that early components of visually-induced ERPs, e.g., P1 or N1, are modulated by stimulus valence. These modulations are thought to reflect the increased attention toward emotional stimuli (Carretié et al., 2004). Emotional valence as well as arousal have been reported to modulate late components known as the late positive potential (LPP) to varying degrees (see Olofsson et al., 2008 for review).

In the frequency domain, valence has more often been associated with transient frontal alpha asymmetry in response to emotional stimuli. Transient alpha asymmetry is characterized by an alpha power difference between the left and right frontal hemispheres during the processing of emotional stimuli (Harmon-Jones et al., 2010). According to asymmetry theory, greater left hemisphere activity during the resting state is associated with emotions involving approach, such as anger and happiness, while greater right hemisphere activity is associated with emotions involving retreat, such as fear (Coan and Allen, 2004; Berkman and Lieberman, 2010; Harmon-Jones et al., 2010). It is important to note that processing of approach-related stimuli in the left hemisphere would be akin to alpha event-related desynchronization (ERD) frontally. One of the most important advantages of using transient alpha asymmetry as a biomarker for valence is that it traditionally has been associated with dynamic emotional responses (Davidson and Fox, 1982; Fox and Davidson, 1988; Wiedemann et al., 1999), (e.g., interpersonal interactions or films), rather than static stimuli such as pictures.

Since the emergence of affective computing, various attempts to classify affective states in the EEG offline have been conducted (Winkler et al., 2010; Makeig et al., 2011; Koelstra et al., 2012). These studies vary greatly in their experimental paradigms, methods used for analyses, and presentation of results rendering a clear state-of-the-art statement rather difficult.

Recently, Brouwer et al. (2015) published recommendations to avoid common pitfalls in the analyses of brain signals that reflect cognitive or affective states. Concisely, these include to identify the state of interest (e.g., cognitive or affective), the expected neurophysiological processes involved, possible confounding factors, good classification practice, insights about features of classification and performance, as well as the added value of employing neurophysiology.

The work presented here seeks to adhere to these with a focus on best practices for conducting and reporting classification results related to brain state classification of affect.

At the same time, the current study aims to determine the validity of the LPP and alpha asymmetry in order to successfully distinguish between unpleasant, neutral, and pleasant emotional states w.r.t. to valence in a healthy adult population.Therefore, an auditory emotion induction paradigm based on the International Affective Digitized Sounds 2nd Edition (IADS-2) database (Bradley and Lang, 2007) was developed in order to induce emotional states. An important goal of the presented work was to determine whether or not the LPP could as well be elicited through auditory stimuli, as it is usually measured with visual stimuli. Furthermore, we intended to evaluate if the auditory LPP can be used for automated affect recognition in a BCI-context.

## 2. Materials and methods

### 2.1. Participants

Twenty-five right-handed healthy participants (12 female; age: 24.46 ± 3.17 years) with normal hearing participated in the study which was approved by the Ethical Review Board of the Medical Faculty, University of Tübingen. Each participant was informed about the purpose of the study and signed informed consent prior to participation. All participants fully completed the experiment. Participants were compensated for their time by 8 Euro/h.

### 2.2. Stimuli

In an attempt to develop an emotion induction paradigm that yields a sufficiently large number of trials and which would easily translate to patient populations, the International Affective Digitized Sounds 2nd Edition (IADS-2) database (Bradley and Lang, 2007) was utilized to induce emotion. Sounds in the database are 6 s long stereo audio recordings of scenic or everyday events. Using IADS-2 allows stimulation via the auditory sensory channel, which tends to be intact in many groups that cannot focus on or otherwise exploit visual information (e.g., patients with cerebral palsy).

The auditory affect induction paradigm consisted of sixty audio files selected from the IADS-2 (see Supplementary Table 3 for full list). All sixty stimuli were categorized into 20 unpleasant events (e.g., vomit, growl, etc.), 20 neutral events (e.g., fan, rooster, etc.), and 20 pleasant events (e.g., baby, laughter, etc.). All sounds were repeated in two separate blocks. Two pseudorandom sequences of consecutive, categorically disjoint sounds were generated for each participant, leading to 120 trials per participant.

### 2.3. Paradigm design

**Figure 1:**
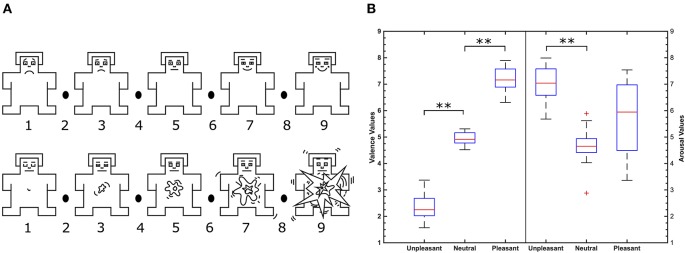
**(A)** Self-assessment manikin in the valence (top) and arousal dimension (bottom). Image is modified from Betella and Verschure (2016). **(B)** Valence (left) and arousal (right) value distributions of IADS-2 sounds selected according to categories. ^**^Indicate significant differences between valence conditions (*p* < 0.01, Wilcoxon test) and ^+^ indicate outliers.

Participants were seated in a comfortable chair approximately 1 m away from a laptop screen with a 15 inch diameter in a quiet room. Participants completed a German version of the Positive Affect Negative Affect Scale (PANAS) (Watson et al., 1988; Krohne et al., 1996) to evaluate current feelings prior to experimentation. All participants were in a normal and relaxed state with no signs of substantial deviations. Standardized audiometry validated binaural hearing capabilities of each participant. The Presentation software kit (Neurobehavioral Systems, Inc.) was used for stimulus presentation. Auditory stimuli were presented via customary computer loudspeakers (Yamaha Co., Hamamatsu, Japan). After attachment of electrodes, task instructions were given. Participants were asked to relax and to actively listen to the sounds presented whilst visually focusing a cross on the laptop screen. After presentation of a 12 s baseline sound, the first sequence of sounds was presented. To assess individual valence and arousal ratings, participants were asked to evaluate each sound after sound-offset with the help of the self assessment manikin (SAM) (Bradley and Lang, 1994) by navigating a 9-point Likert-like scale using the cursor keys on the keyboard. The schematic SAM is shown in Figure 1A. Pressing the up key first confirmed the selection for perceived valence followed by confirmation of the individual arousal rating also marking the end of the trial. The ITI varied randomly between 6 and 14 s in order to maintain participants' task engagement. After presentation of 60 sounds, participants were allowed to relax their eyes and arms for 5 min. The second sequence of sounds was then presented in the same manner lacking the rating step. On average, participants completed the experiment in 2 h including EEG setup.

### 2.4. Data collection and analysis

The electroencephalogram (EEG) along with the vertical and horizontal electrooculogram (EOG) were recorded by active electrodes at 500 Hz sampling frequency and bandpass filtered from 0.1 to 100 Hz (BrainProducts GmbH, Munich, Germany). EEG was recorded from Fp1, Fp2, F3, F4, C3, C4, P3, P4, O1, O2, F7, F8, T7, T8, P7, P8, Fz, Cz, Pz, Tp9, Tp10, Fc1, Fc2, Cp1, Cp2, Fc5, Fc6, Cp5, and Cp6 all referenced to Fcz and grounded against Apz (see Supplementary Figure 1). Continuous EEG was corrected for vertical and horizontal eye movement artifacts (Schlögl et al., 2007). EEG was segmented into 6 s long trials relative to stimulus onset. The data of two participants had to be excluded from analysis due to excessive artifacts leading to *n* = 23 datasets for analysis.

All data analyses were performed offline with a commercial software package (MATLAB 2014b, The MathWorks, Inc., Natick, Massachusetts, United States), FieldTrip (Oostenveld et al., 2011), and custom code. For analysis of event-related potentials (ERPs), EEG was bandpass filtered from 0.1 to 30 Hz with a two-pass Butterworth filter with order 6 and baseline corrected from -0.1 to 0 s relative to stimulus onset. Grand average waveforms were computed for each valence category separately. Waveform differences in the time domain were tested for significance for conditions pleasant vs. neutral, unpleasant vs. neutral, and pleasant vs. unpleasant with a Wilcoxon test and corrected for multiple comparisons by false discovery rate (FDR) (Benjamini and Hochberg, 1995). For analysis of spectral power, pre-processing only included eye movement artifact correction. We computed relative power spectra (Pfurtscheller and Lopes da Silva, 1999) from trial time domain data (0 to 1.4 s relative to stimulus-onset) and 1 s pre-stimulus baseline activity in 1 Hz frequency bins from 1 to 40 Hz by the method of Burg (1967) with a model order of 32.

To analyse if emotional stimuli had an overall effect on power spectra, we conducted an ANOVA with factors participant, power per frequency band delta (1–4 Hz), theta (5–7 Hz), alpha (8–12 Hz), beta (13–29 Hz), and gamma (30–50 Hz) emotional condition (unpleasant, neutral, and pleasant), as well as channel.

### 2.5. Classification of EEG data

Classification of valence categories was evaluated by postulating three binary classification problems: unpleasant vs. neutral, unpleasant vs. pleasant, and pleasant vs. neutral. In the following, classes are occasionally abbreviated with “−” for unpleasant, “0” for neutral, and “+” for pleasant.

#### 2.5.1. Feature extraction and selection

Based on the neurophysiological analysis presented in results, features were extracted from channels Cz, Pz, Cp1, Cp2, Cp5, and Cp6. To reduce the number of features, *R*^2^-values between data and labels was computed for each feature and the features with the highest *R*^2^-values were used for classification (Spüler et al., 2011). Initially, we varied the number of features. Only features that exceeded the mean of all computed *R*^2^-values were taken into account for training the classifier model. On average, 1558 features were used for classification with this setting. As best practice however, we retained only the 100 best scoring features in terms of *R*^2^-values for classification throughout the rest of analyses. As a rule of thumb, the number of features should approximately equal the number of samples (80 in the present study).

#### 2.5.2. Classification

As classifier, we employed a support vector machine (SVM) with a linear kernel (C=1) using libSVM library (Chang and Lin, 2011) which includes fast and efficient implementations of different SVM definitions for classification and regression. In its standard definition, the SVM is the formulation of a geometric and data-driven minimization problem that finds a hyperplane best separating datapoints of two classes under certain conditions (Cortes and Vapnik, 1995). SVMs have been proven to be well suitable for brain state classification especially in the field of BCI research (Lotte et al., 2007). We obtained label predictions as well as prediction probabilities (Platt, 1999; Lin et al., 2007). All performance measures are obtained in a 10-fold cross-validation, i.e., for each participant, feature sets were divided into 10 mutually disjoint training and test sets resulting in 10 sets of 72 training and 8 test instances each.

#### 2.5.3. Performance measures and assessment

To assess classification performance, we investigated three measures: (i) classification accuracy, (ii) area under the curve (AUC) values, and (iii) F1-scores^1^. All three performance measures are different ratios of true positives (TP), true negatives (TN), false positives (FP), and false negatives (FN). In the present study, TPs and TNs are correctly identified valence categories whereas FP and FN are erroneously predicted valence categories of the respective classes of binary classification, (e.g., unpleasant vs. pleasant).

Accuracy as the most common measure for classification performance, is the ratio of TP plus TN divided by the number of test instances. To estimate the quality of classification, obtained accuracy is compared to the chance level of purely random classification. In a binary classification problem with balanced classes in which the number of instances per class is the same, chance level is at 50%. However, the individual significance level threshold of classification accuracy scales with the number of instances per class as well as the number of classes (Müller-Putz et al., 2008). Individual significance level thresholds of accuracy are obtained in permutation tests. Therefore, for each dataset, classification accuracy is repeatedly evaluated in 100 iterations of a 10-fold cross-validation, where on each iteration the class label vector is randomly permuted. Individual significance level thresholds for classification are then obtained by sorting accuracies in an increasing fashion and selecting accuracy values at position 5 for each dataset. If initially computed accuracies exceed obtained thresholds, classification accuracies are significant at *p* < 0.05.

Since permutation tests are accurate but computationally exhaustive, Combrisson and Jerbi (2015) showed that individual significance thresholds can be properly approximated in the context of BCI research, assuming classification errors follow a binominal cumulative distribution. Accordingly for balanced classes, the individual significance level *c*_*i*_(α) at a given significance threshold α is computed by the following MATLAB (The MathWorks, Inc., Natick, Massachusetts, United States) code binoinv(1-α, n, 1/c)*100/n, where *n* is number of samples per class and *c* the number of classes. In the study at hand, for originally computed labeling and assuming a significance threshold of α = 0.05, we obtain an individual significance level *c*_*i*_(α) = 62.5% and for α = 0.01, *c*_*i*_(α) = 70.0%. This approximation is only applicable if all classes are balanced. In different circumstances to properly obtain classification accuracy, permutation tests are recommended.

As a second measure for assessing classification performance, we computed area under the curve (AUC) values from receiver operating characteristic (ROC) curves (Fawcett, 2004). AUC-values are based on true positive and true negative rates computed from thresholds of prediction probabilities of a classifier. The true positive rate is the ratio of TP divided by TP plus FN, whereas the true negative rate is the ratio of TN divided by TN plus FP. To obtain a performance measure that is independent of thresholds, true positive rate and true negative rate are computed by varying thresholds ranging from 0 to 1 in 0.01 steps. The area under the resulting curve is the final AUC-value. As a note for interpretation, AUC-values range from 0 to 1 where 0.5 equals purely random classification, i.e., the classes are statistically identical, values exceeding 0.5 are better than random and vice versa.

As a third measure of classification performance, we computed F1-scores reflecting the harmonic mean of true positive rate and positive predictive value of a binary classifier. Positive predictive value is the ratio of TP divided by TP plus FP. Thus, F1-scores are computed by $\frac{2\cdot \text{TP}}{2\cdot \text{TP}+\text{FP}+\text{FN}}$. F1-scores also range from 0 to 1 with purely random classification at 0.5. Scores exceeding 0.5 are better than random and vice versa. Although F1-scores are claimed to account for class imbalance, these scores are unreliable under certain circumstances (Powers, 2011).

## 3. Results

### 3.1. Stimuli ratings

Emotional categories unpleasant, neutral, and pleasant differed significantly from each other by IADS-2 normative valence as shown in Figure 1B (*p* < 0.01, Wilcoxon test). Significant differences of literature IADS-2 and participants' self reported valence values were not observed in a Wilcoxon test. Participants' self report was correlated with literature IADS-2 valence values. Self reported valence values of all participants highly correlate with literature IADS-2 valence values (*r* = 0.81, *p* < 0.001) verifying the experimental stimuli.

### 3.2. Neurophysiological analysis

**Figure 2:**
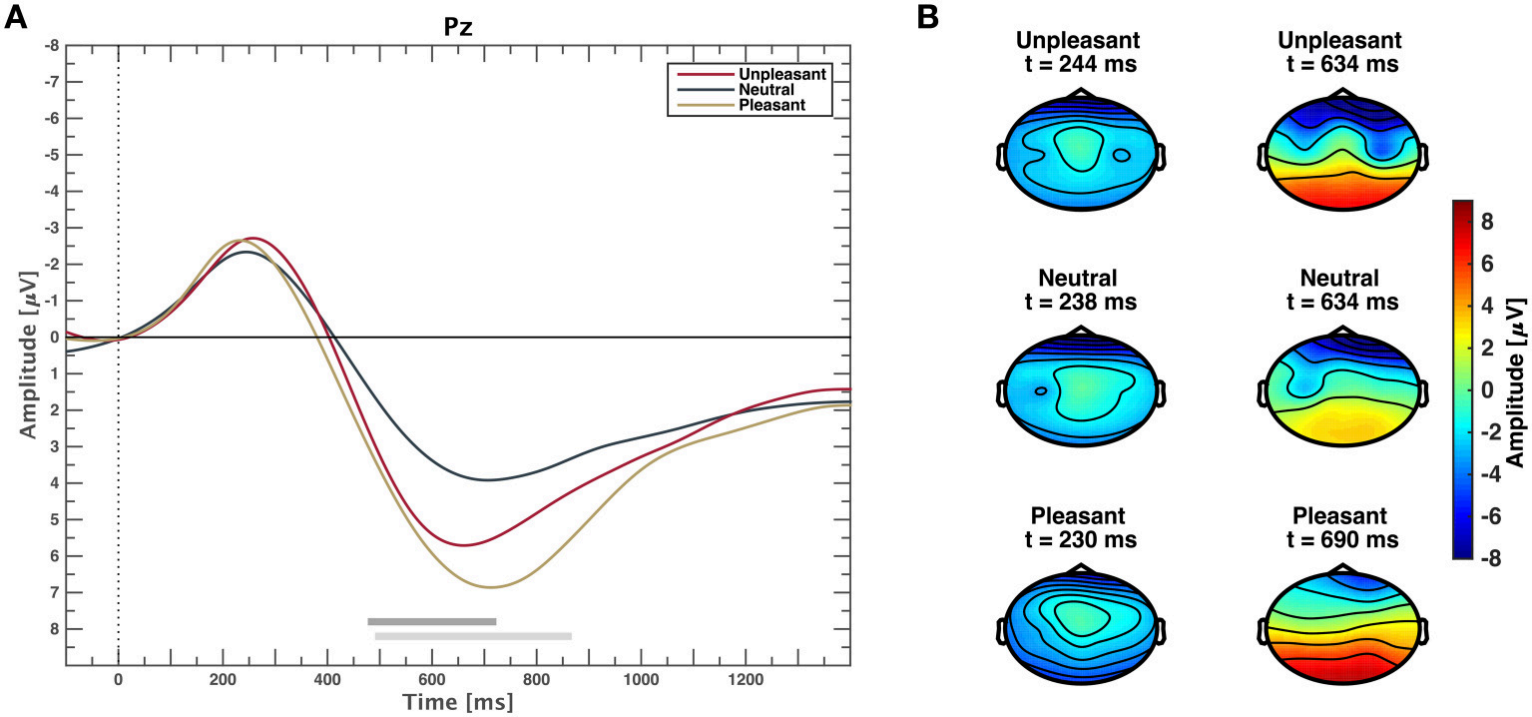
**(A)** Event-related potentials averaged over all participants for unpleasant, neutral, and pleasant stimuli on midline electrode Pz. Gray horizontal bars depict significant differences between neutral and pleasant (light gray) or neutral and unpleasant responses (dark gray), (*p* < 0.05, FDR corrected Wilcoxon test). Differences between unpleasant and pleasant conditions are not significant (*p* > 0.05, FDR corrected Wilcoxon test). **(B)** Scalp plots showing the topographic distribution where grand average responses are minimal (left) and maximal (right) at electrode Pz for unpleasant, neutral, and pleasant stimuli.

The grand average event-related potential time locked to stimulus onset is shown in Figure 2A for each valance category. Clear potentials are visible for responses to all categories. After a negative peak at approximately 200 ms, waveforms of low and high valence stimuli exhibit a stronger positive deflection than neutral valence stimuli that lasts approximately until 1400 ms. Figure 2B depicts scalp plots showing grand average responses on all channels for all categories on time points when amplitudes were minimal and maximal, respectively. Time points for minima and maxima were computed from channel Pz for each emotional condition. After stimulus-onset, amplitudes are more negative in frontal regions across categories. Topographies of responses to unpleasant and pleasant stimuli result in higher positive amplitudes over centro-parietal regions compared to neutral.

Channels Cp1 and Cp2 exhibit the most prominent ERP waveforms with significant responses from 448 to 1400 ms for comparison of categories unpleasant and neutral, as well as pleasant and neutral (see Supplementary Figure 2). On Cp5 and Cp6, only pleasant and neutral responses are significantly different. Marginal interhemispheric waveform differences within the same category at electrode locations Cp1 and Cp2, as well as Cp5 and Cp6 were not significant (*p* > 0.05, FDR corrected Wilcoxon test).

**Figure 3:**
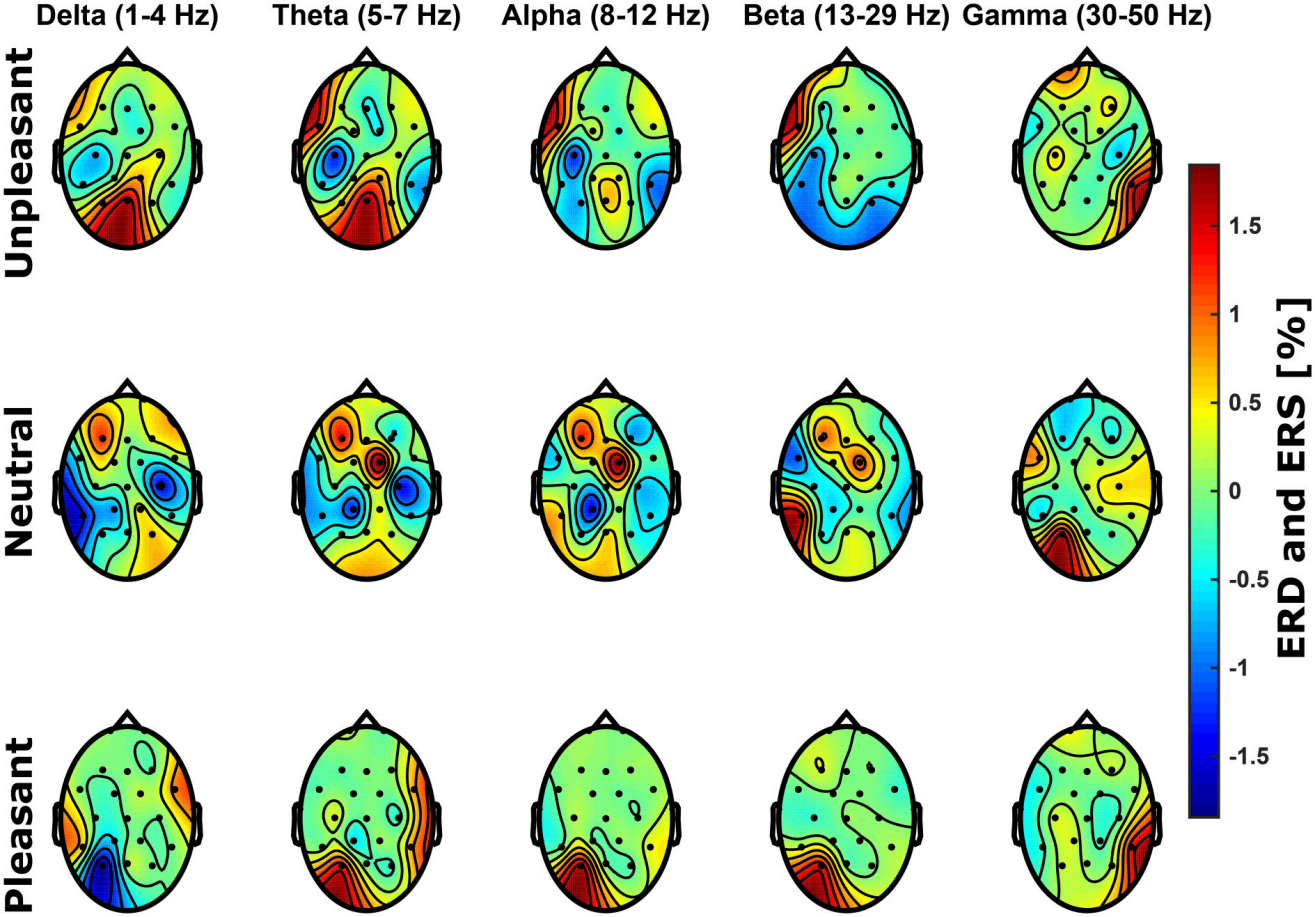
**Scalp topological distributions of grand average event-related de-/synchronization for unpleasant, neutral, and pleasant valence categories relative to baseline spectral power for frequency bands delta (1–4 Hz), theta (5–7 Hz), alpha (8–12 Hz), beta (13–29 Hz), and gamma (30–50 Hz)**.

In the frequency domain, it was expected that the processing of unpleasant sounds results in higher power in the alpha band (8-12 Hz) over right frontal hemispheric regions, whereas power would be elevated over left frontal brain regions for pleasant sounds Davidson (1993, 1998). Figure 3 shows relative spectral power topological distributions of valence categories across frequency bands. There are no significant lateral differences across bands at frontal electrode sites for unpleasant or pleasant condition (*p* > 0.05, FDR corrected Wilcoxon test). Power differences between conditions were not significant (*p* > 0.05, Bonferroni corrected ANOVA).

To investigate the effects of emotional conditions on power spectra, we further conducted an ANOVA with factors participant, frequency band, emotional condition, as well as channel. Significant effects were found for the factors participant, frequency band, emotional condition, as well as channel (*p* < 0.001, Bonferroni corrected).

### 3.3. Time domain classification

Classification was conducted on time domain EEG data where significant differences were observed between conditions on channels Cz, Pz, Cp2, Cp3, Cp4, and Cp5. Three binary classification problems were postulated according to valence categories: unpleasant vs. neutral, unpleasant vs. pleasant, and pleasant vs. neutral. Table 1 depicts average group classification accuracies, AUC-values, and F1-scores. Average group level accuracies and AUC-values for binary classification of unpleasant vs. pleasant and pleasant vs. neutral are significantly above chance. (Individual participant classification results are shown in Supplementary Table 1) Regarding individual classification results in terms of significance levels, only one participant exceeded 62.5% accuracy for unpleasant vs. neutral and unpleasant vs. pleasant. In the classification of pleasant vs. neutral, one participant exceeded the individual significance level 70.0%. With a significance threshold of α = 0.05, we expect on average 1 in 20 participants to exceed the individual significance level by chance.

**Table 1:** **Mean classification accuracies, AUC-values, and F1-scores based on time domain EEG data of channels Cz, Pz, Cp1, Cp2, Cp4, and Cp5 obtained in 10-fold cross-validation**.

	**“−” vs. “0”**	**“−” vs. “+”**	**“+” vs. “0”**
Accuracy	49.99%	53.39%^**^	53.21%^*^
AUC-value	0.49	0.54^**^	0.54^*^
F1-score	0.46	0.51	0.51

Columns indicate classes of respective binary classification problems (“−” unpleasant, “0” neutral, “+” pleasant). Classes are balanced with 40 instances each. Stars indicate significant group differences in a right-tailed t-test against 50 for accuracy and 0.5 for AUC-values and F1-scores with p < 0.05 and p < 0.01, respectively.

To give a valid estimate of individual significance thresholds of classification for the respective performance measure, we conducted permutation tests. Supplementary Table 2 shows individual significance levels at *p* = 0.05 for each participant (for comparison, individual classification performances are shown in Supplementary Table 1). Two participants exceed individual significance levels in all performance measures for the classification of unpleasant vs. neutral, unpleasant vs. pleasant, and pleasant vs. neutral, respectively. One participant slightly exceeded the individual significance level for AUC-values, however not for accuracy nor F1-score. Average accuracy significance thresholds obtained by permutation tests prove the binomial estimate of 62.5% only with deviations lesser than 0.5%.

## 4. Discussion

In this study, we investigated neural responses to emotion-laden sounds by recording EEG, in the context of affective computing. We introduced an auditory emotion induction paradigm also suitable for the study of affect in disabled individuals where visual fixation is absent. Following the dimensional model of emotion, sounds were divided by valence into three categories: unpleasant, neutral, and pleasant. Participants' self report of valence values strongly correlated with literature reported IADS-2 values (*r* = 0.78, *p* < 0.001). Time domain EEG data analysis showed significant grand average waveform differences related to stimulus valence categories. Interhemispheric spectral power differences in the frequency domain related to stimulus valence were not significant. However there was a significant overall effect of stimulus valence to power spectra. Time domain EEG data were subjected to classification using SVM. We found group level significance for the classification of unpleasant vs. pleasant (53.39% accuracy, 0.54 AUC-value) and pleasant vs. neutral (53.21% accuracy, 0.54 AUC-value) conditions. Two participants reached significant individual classification performance in two (unpleasant vs. neutral and unpleasant vs. pleasant) and one condition (pleasant vs. neutral).

### 4.1. Event-related potentials and power spectra

Neurophysiological results in the time domain are consistent with results from earlier studies on affective picture perception (Lang et al., 1997; Cuthbert et al., 2000). Emotional sounds (either unpleasant or pleasant) evoked a larger positive deflection than neutral event-related potentials. After an N2 component, positive deflections begin approximately 400 ms after stimulus-onset and last until approximately 1400 ms for unpleasant and pleasant stimuli. Positive deflections to pleasant stimuli are on average stronger compared to those of unpleasant stimuli, however not significantly. Amplitude differences between neutral and unpleasant or neutral and pleasant conditions are significant over midline and centro-parietal electrode sites. Waveforms at electrodes Cp1 and Cp2 exhibit prolonged positive deflections. Although not as prolonged, these results are in line with late positive potential data of Cuthbert et al. (2000) during the processing of emotion-laden pictures. The observable N2 preceding the LPP is attributed to auditory processing (see Hillyard and Kutas, 1983 for review). An interhemispheric effect of amplitude differences when comparing ERPs of the same condition at Cp1 and Cp2 or Cp5 and Cp6 could not be observed.

Frontal interhemispheric differences in frequency domain power related to stimulus valence reported by Davidson et al. (1990) could not be confirmed. Nonetheless, we found a significant effect of stimulus valence to spectral power confirming the altered brain activity during processing of stimuli. We argue that (not significant) effects in the frequency domain related to hemispheric differences in power and stimulus valence in the present study are attributed to substantial experimental design differences compared to the original study by Davidson et al. (1990). The experimental paradigm in that study employed five 60 s video clips to induce two emotional states (happy and disgust), as well as baseline activity. The first video clip accommodated the participant with the experiment, the subsequent two were clips to induce a positive, and finally two clips to induce a negative emotional condition. Thus, the authors remained with a small number of trials whilst obtaining a relatively large amount of EEG data for analyses. Furthermore, Du and Lee (2014) employed the IADS for emotion induction and analyzed spectral power computed from 18 channel EEG in 30 subjects over a the whole trial length of 6 s. They report significant effects in high alpha (10.86–12.15 Hz), in beta (13.72–29.6 Hz), as well as in low (30.16–38.6 Hz) and high gamma (40–46.75 Hz). We acknowledge the body of affective research in the frequency domain (see Introduction). In the present study however, the total amount of “emotional” EEG of 1.4 s used for spectrum computation seems to be not sufficient to result in significantly measurable power differences in the frequency domain. Our results in the time domain clearly show the LPP as a neurophysiological marker of valence and there is an overall effect of stimulus valence on the power spectrum.

### 4.2. Classification performance assessment

The assessment of classification performance is strikingly influenced by the number of classes, class sizes, as well as class distributions. Thus, it is of utmost importance to clearly report these figures, i.e., two classes with 40 instances each in the present study. Performance metrics such as accuracy, AUC-values, and F1-scores entail a couple of methodological problems. Classification accuracy, as the ratio between correctly classified instances and all instances, is probably the most prominent measure for classification quality assessment. In a generic two-, three-, or *n*-class classification problem, a straight-forward approach is to evaluate classification accuracy in a 10-fold cross-validation and investigate the deviation of obtained accuracy from random classification, i.e., the so called chance level at 50, $33.\bar{3}$, or $\frac{100}{n}$%, respectively. The most severe problem is that this computation of chance level is only valid for balanced classes, i.e., the number of instances per class is the same for all classes. Complying with this prerequisite, accuracy computed by 10-fold cross-validation is a valid measure to estimate classification performance against the chance level. As will be outlined in the following, the performance assessment in brain state classification on a participant level requires further measures. From a theoretical point of view, individual significance thresholds in classification only hold for an unlimited number of training and testing instances (Müller-Putz et al., 2008). Although this limitation is commonly accepted in the machine learning community, it seems not well-established in interdisciplinary fields such as affective computing where studies are especially prone to a small number of trials. To properly estimate individual significance thresholds of classification, we strongly encourage to conduct permutation tests. These tests are not only independent of the performance measure, but also independent of class distributions. Since permutation tests can be time consuming, we also suggest to compute individual chance levels according to Combrisson and Jerbi (2015). Nonetheless, we want to emphasize that this approach is only valid for accuracy and if classes are balanced. In this regard, we strongly encourage to design studies such that trials are equal across experimental conditions. If class distributions are skewed however, (e.g., due to technical failures or processing steps), we suggest to assess classifier performance by AUC-values. Statistics for group level analyses are similar to accuracy. On the participant level however, permutation tests are again a must. The interested reader is directed to the introductory article by Fawcett (2006) for more information on AUC-values. The main disadvantage of F1-scores is that true negatives are neglected in their computation. Thus, F1-scores are known to be unreliable under certain circumstances (Powers, 2011). In terms of statistical analyses, the same policy as for AUC-values applies.

### 4.3. Classification

For single trial classification of time domain LPP data, we could show that classification of unpleasant vs. pleasant and pleasant vs. neutral was possible with accuracies and AUC-values above chance at group level. We followed a data processing cascade common to BCI practices. Fast feature reduction and selection based on *R*^2^-values along with binary SVM classification yielded best results with 100 features and a linear kernel. However, we only reached average accuracies of about 53%, which are only significant at group level and not at participant-level. Thereby the application of machine learning methods merely serves as a confirmation that there are valence-related effects in the data, but that these effects are too small, so that the application for automatic affect recognition is not feasible with the presented approach. Although the reference electrode Fcz may seem unusual in an ERP analysis, we followed a standard electrode montage layout provided by BrainProducts for it showed best results as compared to re-referencing the data. Furthermore in the time domain, re-referencing as an arithmetic operation can be easily learned during linear SVM model training and is therefore negligible for classifier training and classification prediction performance.

In comparison with other studies, Koelstra et al. (2012) conducted emotion induction by videos and also reported significant above chance level classification of EEG data regarding positive and negative valence. With an accuracy of 57.6% they obtained results in a similar range as ours although a bit higher. However, these results are not directly comparable, as the classes were not evenly distributed, which stresses the importance of using measures like AUC to compare results with different class distributions across studies. In the present study, classification was also done solely in the time domain using the LPP while Koelstra et al. (2012) employed power spectral features. As we only classified validated features of neurophysiological emotional processing, power spectra were not classified since our findings regarding interhemispheric frontal power difference related to emotional processing were not significant. In this point, our results are in contrast to Pan et al. (2013) who showed successful classification of 74.77% accuracy in the preference between liking and disliking of music listening employing frontal power spectral features computed from 30 s frontal 2-channel EEG in 12 subjects. However, binary preferences in music listening in terms of liking and disliking are not directly comparable to valence in relation to dimensional emotion theory (Russel, 1980; Russell and Barrett, 1999). Nevertheless, the classification performance is currently too low to be feasible for automatic affect recognition for a working application. This shows that besides better strategies for reporting and assessing classification performance, also better methods for EEG signal processing are needed to reduce the amount of noise in the data and improve affect classification.

Research on affect classification requires to answer two linked questions: Firstly, are there significant differences regarding stimulus valence (arousal or discrete emotion) and respective electrophysiological changes in the time and/or frequency domain? Secondly, can a classifier model successfully be trained and discern the stimulus valence from those statistically validated electrophysiological changes in the time and/or frequency domain of unseen data (on single or multiple trial basis)? As outlined, many studies have sought to answer the first question, yet the second question has not been investigated. We have identified stimulus duration as an interesting topic for conducting further research, e.g., Diamond and Zhang (2016) found significant oscillatory effects in the gamma band that mediate emotional processing of speech even for word stimuli even as short as 295 ms yet have not addressed whether accurate classification of valence can be obtained by those spectral features. Based on the results of the current study, the “emotional” EEG length of 1.4 s is still insufficient for classification solely based on spectral features. Given the discrepancies of classification among the classification studies, future research could address what the optimal time length of EEG data is for classifying the emotional valence more accurately and reliably.

## 5. Conclusion

Neural responses to emotion-laden sounds were validated in the time- yet not in the frequency domain. The visually evoked LPP as a neurophysiological marker of emotional processing was investigated. Interhemispheric frontal differences in spectral power were not significant. Measures regarding good classification practices were discussed. Following a BCI processing cascade, classification results of LPP for valence were significantly above chance at group level.

## Author contributions

DH, EB, TM, and NB conceptualized the problem and the study design. DH, EB, TM, NB, WR, and MS discussed analyses. DH implemented and conducted analyses. NB, WR, and MS supervised the work. DH, EB, TM, NB, WR, and MS wrote the paper.

## Funding

This study was funded by the Seventh Framework Programme (FP7) - EU Contract: FP7-ICT-2011-7-287774 and supported by the WissenschaftsCampus, Tübingen as well as the Deutsche Forschungsgesellschaft (DFG, Koselleck to NB and SP 1533/2-1). We acknowledge support by Deutsche Forschungsgemeinschaft and Open Access Publishing Fund of University of Tübingen.

### Conflict of interest statement

The authors declare that the research was conducted in the absence of any commercial or financial relationships that could be construed as a potential conflict of interest.

## Footnotes

^1^The source code for feature reduction, classification by SVM, as well as the computations for accuracies, AUC-values, and F1-scores is freely available at https://github.com/dthettich/BSClassify

## Supplementary material

Supplementary Table 1 **Individual classification accuracies, AUC-values, and F1-scores based on time domain EEG data of channels Cz, Pz, Cp1, Cp2, Cp4, and Cp5 obtained in 10-fold cross-validation**. Columns indicate classes of respective binary classification problems (“−” unpleasant, “0” neutral, “+” pleasant). Classes are balanced with 40 instances each. Stars indicate significant group differences in a right-tailed *t*-test against 50 for accuracy and 0.5 for AUC-values and F1-scores with *p* < 0.05 and *p* < 0.01, respectively.

Supplementary Table 2 **Individual significance levels of classification at significance threshold α = 0.05 obtained by permutation tests for the performance measures accuracy, AUC-value, and F1-score based on time domain EEG data of channels Cz, Pz, Cp1, Cp2, Cp4, and Cp5 in 100 iterations**. Columns indicate classes of respective binary classification problems (“−” unpleasant, “0” neutral, “+” pleasant). Classes are balanced with 40 instances each.

Supplementary Table 3 **IADS-2 sound ids and respective valence/arousal values for each emotional category**.

Supplementary Figure 1 **Scalp topography of electrode locations Fp1, Fp2, F3, F4, C3, C4, P3, P4, O1, O2, F7, F8, T7, T8, P7, P8, Fz, Cz, Pz, Tp9, Tp10, Fc1, Fc2, Cp1, Cp2, Fc5, Fc6, Cp5, and Cp6 all referenced to Fcz and grounded against Apz**.

Supplementary Figure 2 **Event-related potentials averaged over all participants for unpleasant, neutral, and pleasant stimuli on electrodes Cp1 and Cp2 (A) as well as Cp5 and Cp6 (B)**. Gray horizontal bars depict significant differences between neutral and pleasant (light gray) or neutral and unpleasant responses (dark gray), (*p* < 0.05, FDR corrected Wilcoxon test). Differences between unpleasant and pleasant conditions are not significant (*p* > 0.05, FDR corrected Wilcoxon test). There are no significant differences between event-related potentials measures at opposite electrodes (*p* > 0.05, FDR corrected Wilcoxon test).
