# Supplementary material for: EEG Responses to Auditory Stimuli for Automatic Affect Recognition
Source: Front Neurosci. 2016 Jun 10;10:244. doi: 10.3389/fnins.2016.00244 (PMC4901068; doi:10.3389/fnins.2016.00244)
Supplement: Supplementary Table 1 — Individual classification accuracies, AUC-values, and F1-scores based on time domain EEG data of channels Cz, Pz, Cp1, Cp2, Cp4, and Cp5 obtained in 10-fold cross-validation. Columns indicate classes of respective binary classification problems (“−” unpleasant, “0” neutral, “+” pleasant). Classes are balanced with 40 instances each. Stars indicate significant group differences in a right-tailed t-test against 50 for accuracy and 0.5 for AUC-values and F1-scores with p < 0.05 and p < 0.01, respectively. [file Table1.PDF]

Supplementary Table 1. Individual classification accuracies, AUC-values, and F1-scores based on time domain EEG data of channels Cz, Pz, Cp1, Cp2, Cp4, and Cp5 obtained in 10-fold cross-validation. Columns indicate classes of respective binary classification problems ('-' unpleasant, '0' neutral, '+' pleasant). Classes are balanced with 40 instances each. Stars indicate significant group differences in a right-tailed t-test against 50 for accuracy and 0.5 for AUC-values and F1-scores with $p < 0.05$ and $p < 0.01$, respectively.

	**'-' vs. '0'**			**'-' vs. '+'**			**'+' vs. '0'**		
**Participant**	**Accuracy**	**AUC**	**F1-Score**	**Accuracy**	**AUC**	**F1-Score**	**Accuracy**	**AUC**	**F1-Score**
S01	63.75 %	0.65	0.64	67.50 %	0.73	0.68	46.25 %	0.49	0.46
S02	54.82 %	0.52	0.51	59.29 %	0.54	0.56	55.00 %	0.54	0.57
S03	52.50 %	0.55	0.53	51.25 %	0.49	0.49	52.50 %	0.52	0.49
S04	53.75 %	0.52	0.51	56.25 %	0.58	0.53	51.25 %	0.46	0.48
S05	56.25 %	0.58	0.56	53.75 %	0.57	0.53	73.75 %	0.82	0.71
S06	45.00 %	0.45	0.39	57.50 %	0.59	0.60	55.00 %	0.54	0.50
S07	46.25 %	0.47	0.38	46.25 %	0.48	0.47	57.50 %	0.56	0.57
S08	41.25 %	0.42	0.30	50.00 %	0.49	0.43	57.50 %	0.59	0.60
S09	46.25 %	0.54	0.48	40.00 %	0.40	0.33	60.00 %	0.59	0.57
S10	47.50 %	0.49	0.40	48.75 %	0.54	0.45	57.50 %	0.62	0.56
S11	47.50 %	0.45	0.49	60.00 %	0.58	0.57	48.75 %	0.49	0.44
S12	47.50 %	0.47	0.45	56.25 %	0.60	0.53	58.75 %	0.58	0.55
S13	55.00 %	0.51	0.54	50.00 %	0.50	0.44	62.50 %	0.65	0.58
S14	47.50 %	0.41	0.30	48.75 %	0.49	0.37	46.25 %	0.51	0.38
S15	50.00 %	0.49	0.39	51.25 %	0.49	0.51	48.75 %	0.43	0.48
S16	43.75 %	0.42	0.43	60.00 %	0.60	0.53	47.50 %	0.52	0.50
S17	51.25 %	0.49	0.52	53.75 %	0.57	0.49	43.75 %	0.41	0.33
S18	43.75 %	0.42	0.38	53.75 %	0.52	0.60	45.00 %	0.46	0.41
S19	57.50 %	0.53	0.55	50.00 %	0.47	0.51	48.75 %	0.49	0.42
S20	43.75 %	0.48	0.40	53.75 %	0.51	0.51	60.00 %	0.60	0.60
S21	48.75 %	0.46	0.44	53.75 %	0.47	0.53	41.25 %	0.45	0.41
S22	52.50 %	0.54	0.47	57.50 %	0.58	0.54	55.00 %	0.55	0.56
S23	53.75 %	0.46	0.51	48.75 %	0.52	0.49	51.25 %	0.53	0.55
**Mean**	49.99 %	0.49	0.46	53.39 % **	0.54 **	0.51	53.21 % *	0.54 *	0.51
