# Supplementary material for: EEG Responses to Auditory Stimuli for Automatic Affect Recognition
Source: Front Neurosci. 2016 Jun 10;10:244. doi: 10.3389/fnins.2016.00244 (PMC4901068; doi:10.3389/fnins.2016.00244)
Supplement: Supplementary Table 2 — Individual significance levels of classification at significance threshold α = 0.05 obtained by permutation tests for the performance measures accuracy, AUC-value, and F1-score based on time domain EEG data of channels Cz, Pz, Cp1, Cp2, Cp4, and Cp5 in 100 iterations. Columns indicate classes of respective binary classification problems (“−” unpleasant, “0” neutral, “+” pleasant). Classes are balanced with 40 instances each. [file Table2.PDF]

Supplementary Table 2. Individual significance levels of classification at significance threshold $\alpha = 0.05$ obtained by permutation tests for the performance measures accuracy, AUC-value, and F1-score based on time domain EEG data of channels Cz, Pz, Cp1, Cp2, Cp4, and Cp5 in 100 iterations. Columns indicate classes of respective binary classification problems ('-' unpleasant, '0' neutral, '+' pleasant). Classes are balanced with 40 instances each.

	**'-' vs. '0'**			**'-' vs. '+'**			**'+' vs. '0'**		
**Participant**	**Accuracy**	**AUC**	**F1-Score**	**Accuracy**	**AUC**	**F1-Score**	**Accuracy**	**AUC**	**F1-Score**
S01	61.25 %	0.63	0.61	63.75 %	0.67	0.64	61.25 %	0.64	0.60
S02	61.07 %	0.65	0.61	63.21 %	0.65	0.63	63.75 %	0.65	0.62
S03	63.75 %	0.66	0.65	62.50 %	0.66	0.63	63.75 %	0.63	0.63
S04	63.75 %	0.63	0.65	63.75 %	0.63	0.62	60.00 %	0.65	0.60
S05	62.50 %	0.63	0.62	65.00 %	0.68	0.64	61.25 %	0.62	0.61
S06	63.75 %	0.62	0.64	63.75 %	0.64	0.65	65.00 %	0.66	0.63
S07	63.75 %	0.69	0.62	66.25 %	0.65	0.65	63.75 %	0.64	0.63
S08	62.50 %	0.63	0.62	61.25 %	0.63	0.61	61.25 %	0.66	0.63
S09	65.00 %	0.65	0.63	63.75 %	0.65	0.66	62.50 %	0.64	0.63
S10	60.00 %	0.62	0.59	63.75 %	0.66	0.63	61.25 %	0.63	0.64
S11	63.75 %	0.64	0.64	61.25 %	0.67	0.62	63.75 %	0.64	0.63
S12	61.25 %	0.65	0.62	62.50 %	0.66	0.63	62.50 %	0.64	0.64
S13	62.50 %	0.60	0.64	60.00 %	0.62	0.62	62.50 %	0.64	0.63
S14	65.00 %	0.67	0.66	62.50 %	0.67	0.64	63.75 %	0.66	0.62
S15	60.00 %	0.63	0.61	60.00 %	0.64	0.61	62.50 %	0.64	0.63
S16	63.75 %	0.65	0.64	65.00 %	0.66	0.64	61.25 %	0.63	0.61
S17	65.00 %	0.62	0.64	62.50 %	0.67	0.64	62.50 %	0.66	0.64
S18	58.75 %	0.62	0.60	62.50 %	0.62	0.62	62.50 %	0.63	0.62
S19	60.00 %	0.66	0.59	61.25 %	0.64	0.60	62.50 %	0.64	0.62
S20	63.75 %	0.65	0.63	63.75 %	0.65	0.63	62.50 %	0.65	0.62
S21	65.00 %	0.64	0.66	62.50 %	0.67	0.64	62.50 %	0.67	0.62
S22	62.50 %	0.60	0.63	61.25 %	0.64	0.61	62.50 %	0.64	0.65
S23	63.75 %	0.65	0.64	62.50 %	0.62	0.63	65.00 %	0.63	0.66
**Mean**	62.71 %	0.64	0.63	62.80 %	0.65	0.63	62.61 %	0.64	0.63
