# Supplementary material for: EEG Responses to Auditory Stimuli for Automatic Affect Recognition
Source: Front Neurosci. 2016 Jun 10;10:244. doi: 10.3389/fnins.2016.00244 (PMC4901068; doi:10.3389/fnins.2016.00244)
Supplement: Supplementary Table 3 — IADS-2 sound ids and respective valence/arousal values for each emotional category. [file Table3.PDF]

Supplementary Table 3. IADS-2 sound ids and respective valence/arousal values for each emotional category.

Unpleasant			Neutral			Pleasant		
**Id**	**Val.**	**Aro.**	**Id**	**Val.**	**Aro.**	**Id**	**Val.**	**Aro.**
106	1.57	5.68	102	4.52	2.88	110	6.31	3.36
115	1.68	6.07	120	4.52	4.03	112	6.62	3.36
244	1.68	6.31	170	4.63	4.12	151	6.81	4.18
255	1.93	6.39	246	4.68	4.35	172	6.82	4.46
260	2.01	6.57	262	4.72	4.41	200	6.84	4.47
276	2.04	6.59	322	4.83	4.42	202	6.94	4.51
278	2.04	6.82	358	4.83	4.60	220	6.94	4.95
279	2.06	6.87	364	4.83	4.60	226	6.97	5.42
284	2.08	6.91	368	4.86	4.65	311	7.00	5.87
286	2.16	7.03	373	4.88	4.65	360	7.12	5.89
288	2.34	7.05	376	4.95	4.65	365	7.20	6.00
289	2.42	7.08	410	5.01	4.75	716	7.28	6.03
296	2.44	7.10	425	5.09	4.79	726	7.40	6.32
420	2.61	7.27	627	5.09	4.87	809	7.44	6.44
424	2.65	7.39	698	5.15	4.91	810	7.51	6.85
624	2.71	7.77	700	5.18	4.97	811	7.64	7.10
703	2.82	7.88	701	5.19	5.15	813	7.65	7.12
711	2.89	7.95	722	5.20	5.41	815	7.67	7.13
712	3.08	7.98	723	5.26	5.62	817	7.78	7.15
719	3.37	7.99	728	5.31	5.89	820	7.90	7.54
**Mean**	2.34	7.04	**Mean**	4.94	4.69	**Mean**	7.19	5.71
