# Supplementary figures and images for: EEG Responses to Auditory Stimuli for Automatic Affect Recognition
Source: Front Neurosci. 2016 Jun 10;10:244. doi: 10.3389/fnins.2016.00244 (PMC4901068; doi:10.3389/fnins.2016.00244)

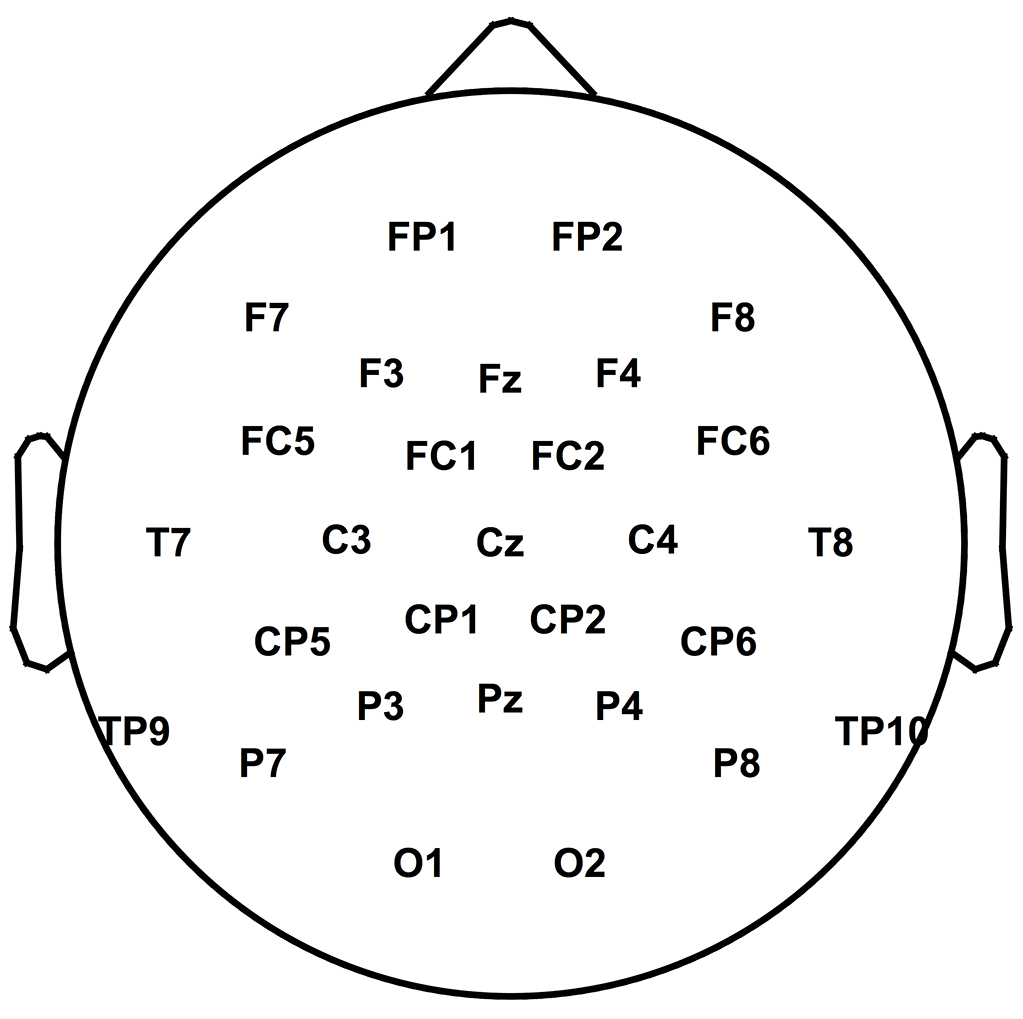

Supplement: Supplementary Figure 1 — Scalp topography of electrode locations Fp1, Fp2, F3, F4, C3, C4, P3, P4, O1, O2, F7, F8, T7, T8, P7, P8, Fz, Cz, Pz, Tp9, Tp10, Fc1, Fc2, Cp1, Cp2, Fc5, Fc6, Cp5, and Cp6 all referenced to Fcz and grounded against Apz. [file Image1.TIFF]

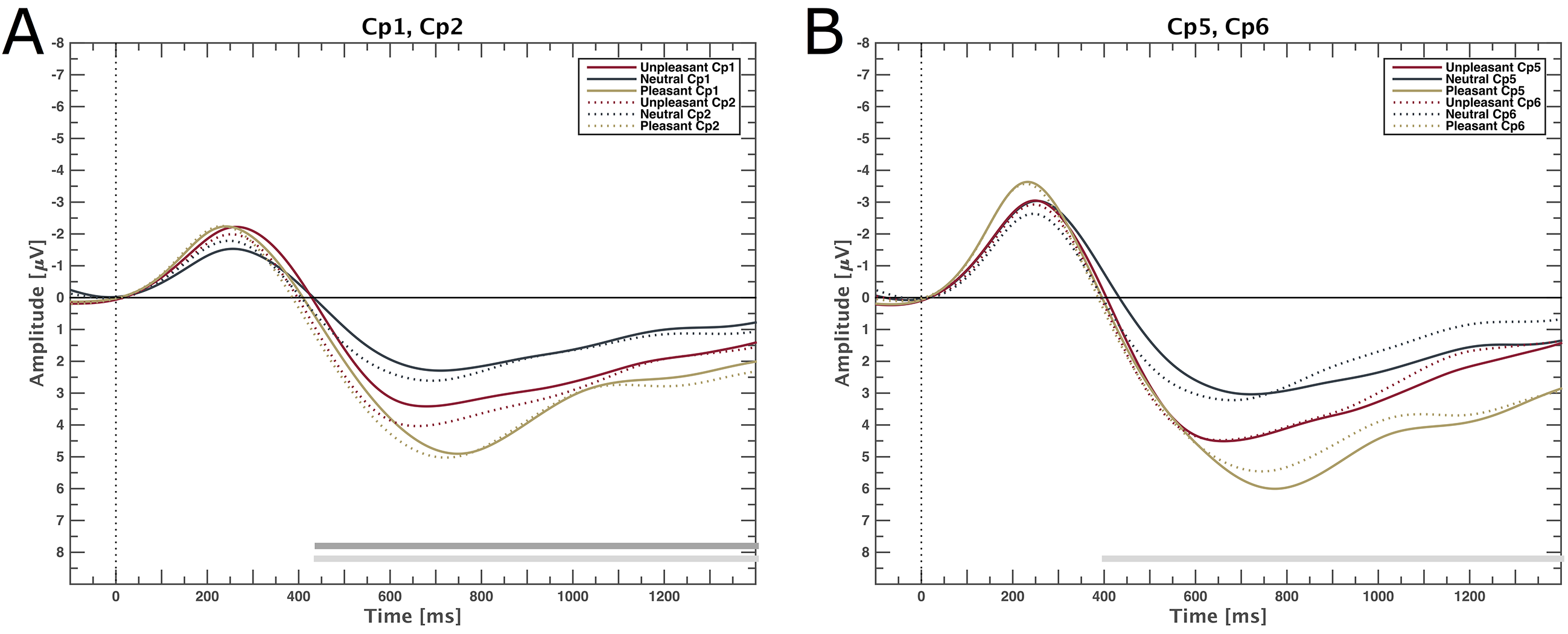

Supplement: Supplementary Figure 2 — Event-related potentials averaged over all participants for unpleasant, neutral, and pleasant stimuli on electrodes Cp1 and Cp2 (A) as well as Cp5 and Cp6 (B). Gray horizontal bars depict significant differences between neutral and pleasant (light gray) or neutral and unpleasant responses (dark gray), (p < 0.05, FDR corrected Wilcoxon test). Differences between unpleasant and pleasant conditions are not significant (p > 0.05, FDR corrected Wilcoxon test). There are no significant differences between event-related potentials measures at opposite electrodes (p > 0.05, FDR corrected Wilcoxon test). [file Image2.TIFF]
